# Factors Associated with Intention to Resign among Medical Residents during the COVID-19 Pandemic in Japan: A Cross-sectional Study

**DOI:** 10.31662/jmaj.2023-0004

**Published:** 2023-07-04

**Authors:** Takako Morita, Saki Muroya, Osamu Takahashi, Sachiko Ohde

**Affiliations:** 1 Graduate School of Public Health and Clinical Epidemiology St. Luke’s International University, Tokyo, Japan

**Keywords:** Intention to resign, COVID-19, Medical resident, Mental health, Public health

## Abstract

**Introduction:**

The coronavirus disease 2019 (COVID-19) pandemic has significantly affected healthcare workers’ mental health worldwide, leading to the intention to resign. Japanese medical residents were no exception to the impact of COVID-19. This study aimed to illustrate how the COVID-19 pandemic affected medical residents’ intention to resign.

**Methods:**

A cross-sectional study was conducted for Postgraduate Year (PGY)1-5 medical residents in Japan using an internet survey from March 11 to March 18, 2021. During this survey, the Japanese government declared a second-time state of emergency on January 7, 2021, and all restrictions were expanded nationwide until March 21, 2021. Medical residents were categorized into two groups (intention to resign group [IR] or no intention to resign group [NIR]) based on their intention to resign. Multivariate logistic regression analysis was conducted to elucidate the risk factors for the intention to resign.

**Results:**

354 medical residents were enrolled in this study (response rate: 40.2%). Ninety-two medical residents (26.0 %) were categorized into IR and 262 (74.0%) into NIR. According to multivariate logistic regression analysis, those who did not have mental support at their hospital had 2.95 times greater chance of intending to resign (Adjusted odds ratio [AOR] = 2.95, 95% confidence interval (CI) [1.42-6.15]). Medical residents who engaged in patients with COVID-19 (AOR = 2.13, 95% Cl [1.08-4.18]) and PGY5 (AOR = 3.38, 95% Cl [1.51-7.56]) had a higher likelihood of intending to resign among residents in all PGY.

**Conclusions:**

One in four medical residents intended to resign during the COVID-19 pandemic. Particularly, PGY5 and medical residents who treated patients with COVID-19 were found at risk of having the intention to resign. At times of crisis, such as the COVID-19 epidemic, establishing a mental health support system that targets high-risk residents is critical.

## Introduction

The coronavirus disease 2019 (COVID-19) pandemic significantly affected and drastically changed our lives, including changes to our daily routines through lockdowns ^(1), (2)^. Medical students and healthcare workers (HCWs) also received a tremendous burden due to COVID-19. During the COVID-19 pandemic, medical students experienced increased stress and anxiety due to oppressed responsibilities and lack of resources ^(3)^. HCWs have performed unusual tasks with inadequate medical resources and educational opportunities for infection prevention. For this reason, HCWs had to take care of patients with COVID-19 with the fear of infecting their families ^(4)^. Medical residents were also forced to change their training programs or missed learning opportunities. They had to treat patients with COVID-19 under the downsized medical care system and the lack of infection protection resources such as Personal Protective Equipment (PPE) ^(5)^. In this context, a meta-analysis reported that the prevalence of anxiety, depression, and insomnia among HCWs who engaged in COVID-19-related tasks were 23.2%, 22.8%, and 38.9%, respectively ^(6)^. Indeed, HCWs had a higher prevalence of mental health distress than the general population ^(7)^. Incidentally, 14.6% of medical residents, regardless of whether they directly cared for patients with COVID-19 or not, reported reconsidering their profession due to the COVID-19 pandemic ^(8)^. Additionally, 15.4% of nursing staff in Japan reported leaving their jobs in the first wave of the epidemic ^(9)^.

In Japan, young medical doctors in PGY 1-2 are trained in the national standardized two-year residency program, where they rotate through different medical departments (internal medicine, emergency, community medicine, surgery, pediatrics, obstetrics and gynecology, and psychiatry), and afterward, step up to the advanced specialty training in PGY 3-5 ^(10)^. The Minimum Wage Act sets PGY 1 and PGY 2’s salary, and the residency guidelines guarantee their housing environment ^(10)^.

Japanese medical residents are also facing mental health distress issues. During the COVID-19 pandemic, quite a few medical residents in Japan also lost the chance to proceed with scheduled training and were forced to work in the emergency department ^(11), (12)^. Nearly 20% of medical residents in Japan reported depression ^(13)^. Previous reports showed that mental health distress was related to excessive working hours, individual stress-coping ability, and individual job quality and satisfaction ^(14), (15), (16)^. Notably, postgraduate year (PGY) 1 has been vulnerable to depression due to the change in environment from student to professional life ^(17)^. However, little is known about the factors contributing to medical residents intending to resign under COVID-19. Therefore, this study aimed to illustrate how the COVID-19 pandemic affected medical residents’ intention to resign.

## Materials and Methods

### Study design and participants

A cross-sectional study was conducted at PGY 1-5, using an internet survey from March 11 to March 18, 2021. Contents of the survey items were developed through reviews of previous literature ^(18), (19), (20), (21), (22), (23), (24), (25), (26), (27), (28), (29)^. Participants were recruited from the research panel provided by a commercial survey company (PLAMED plus Co., Ltd, Tokyo, Japan). Obtained data included gender, age, PGY, lifestyle (working hours and sleeping hours), specialty, affiliated hospital (location and the number of hospital beds), presence of mental health support at their hospital, intention to resign from work, if they engaged in the treatment of patients with COVID-19, the number of patients they treated with patients with COVID-19, change in communication frequency with supervising physicians, and interest in public health. The data on mental health at their hospital did not include whether they were active or not. The contents of the survey items are shown in the Supplemental Materials. During this survey, the Japanese government declared a second-time state of emergency on January 7, 2021, and all restrictions were expanded nationwide until March 21, 2021.

Participants answered the online questionnaire using their cell phones or personal computer. After data collection, participants were anonymized for subsequent analyses. Each participant was compensated with a gift card of JPY 1,000 (approximately USD 10.00).

### Statistical analysis

Participants were categorized into two groups based on their intention to resign. Participants who answered “I sometimes have thought about it” or “I am thinking about it right now” were categorized into the intention to resign group (IR); participants who answered “I never thought about it” or “I have thought about it only once” were into the no intention to resign group (NIR).

Descriptive analyses were employed to characterize participants between IR and NIR. Categorical variables were expressed as counts and percentages. Continuous variables were expressed as mean and standard deviation. Univariate analysis was conducted to clarify relevant differences between IR and NRI. Chi-squared test was used for comparing categorical variables. Student t-test was used for continuous variables. To select the final predictors, all candidate predictors for which *P*-value was <0.2 in univariate analysis, along with gender, were selected and included in a multivariate logistic regression model. Multivariate logistic analysis models were fitted to calculate the adjusted odd ratio (AOR) with 95% confidence interval (CI) for IR, adjusted with participating characteristics and affiliation conditions. Characteristics of participants include gender, PGY, sleeping hours, the number of patients with COVID-19 they treated, communication frequency with supervising physicians, and an interest in public health. Affiliation conditions include the number of hospital beds and the presence of mental support. The significance level was defined at *P* < 0.05; all tests were two-tailed. STATA version 17.0 (StataCorp., College Station, Tx, USA) performed the statistical analysis.

### Ethical statement

This study was conducted per the Declaration of Helsinki and approved by the Research Ethics Committee of St. Luke’s International Hospital (approval code: 20-R216). Informed consent was through a form on the questionnaire website.

## Results

As of March 2021, 880 medical residents at PYG 1-5 were requested to participate in this survey. A total of 354 medical residents (response rate: 40.2%) enrolled in this study. Table 1 shows their demographic information. 279 of 354 respondents (78.8%) were male, and 296 (83.6%) age under 30 years old. The breakdown of PYG [n (%)]: PGY1, 93 (37.2%); PGY2, 85 (42.5%); PGY3, 69 (46.0%); PGY4, 51 (34.0%); and PGY5, 56 (43.1%). Medical residents engaged in treating patients with COVID-19 were 76.8%. Throughout the COVID-19 pandemic, 52.0% of the medical residents newly developed an interest and concern for public health. Half of the participants recognized mental health support at their hospital.

**Table 1.** The Demographic of Medical Residents Who Had the Intention to Resign during the COVID-19 Pandemic.

	Total (n = 354)	IR^a^ (n = 92)	NIR ^b^ (n = 262)	*P-*value
Gender [n (%)]				0.880
Male	279	72 (25.8)	207(74.2)
Female	75	20 (26.7)	55 (73.3)
Age [n (%)]				0.310
24-25	47	9 (19.2)	38 (80.9)
26-30	249	62 (24.9)	187 (75.1)
31-35	38	13 (34.2)	25 (65.8)
36-40	18	7 (38.9)	11 (61.1)
41-45	2	1 (50.0)	1 (50.0)
PGY [n (%)]				0.003*
PGY 1	93	17 (18.3)	76 (81.7)
PGY 2	85	17 (20.0)	68 (80.0)
PGY 3	69	16 (23.2)	53 (76.8)
PGY 4	51	17 (33.3)	34 (66.7)
PGY 5	56	25 (44.6)	31 (55.4)
Working hours [Mean (SD)]	50.8 (18.7)	52.0 (17.3)	50.4 (19.2)	0.469
Sleeping hours [Mean (SD)]	6.3 (0.92)	6.2 (1.03)	6.38 (0.88)	0.168
Location of the hospital^c^ [n (%)]				0.421
Urban area	239	59 (24.7)	180 (75.3)
Others	115	33 (28.7)	82 (71.3)
Number of hospital beds [n (%)]				0.052
<300	40	13 (32.5)	27 (67.5)
300-700	222	48 (21.6)	174 (78.4)
≥700	92	31 (33.7)	61 (66.3)
Presence of mental health support at the hospital [n (%)]				<0.001*
Yes	183	36 (19.7)	147 (80.3)
No	53	25 (47.2)	28 (52.8)
Do not know	118	31 (26.3)	87 (73.7)
Treated patients with COVID-19 [n (%)]				0.070
Yes	272	77 (28.3)	195 (71.7)
No	82	15 (18.3)	67 (81.7)
Number of treating patient with COVID-19 [mean (SD)]	8.71 (26.1)	9.03 (12.3)	8.58 (29.9)	0.900
Communication frequency with supervising physicians [n (%)]				0.073
Increased	41	14 (34.2)	27 (65.9)
Decreased	97	31 (32.0)	66 (68.0)
No change	216	47 (21.8)	169 (78.2)
In the face of this COVID-19 pandemic, have you become interested or concerned about public health? [n (%)]				0.012*
Yes	184	39 (21.2)	145 (78.8)
already been interested in	56	23 (41.1)	33 (58.9)
No	114	30 (26.3)	84 (73.7)
Specialized Clinical Department ^d^				0.901
Department of Medicine	94	31 (33.0)	63 (67.0)
Department of Surgery	81	26 (32.1)	55 (67.9)

*P < 0.05 Abbreviations: COVID-19, coronavirus disease 2019; SD, Standard deviation; PGY, postgraduate year ^a^ IR, intention to resign group, have the intention of resigning either right away/sometime soon ^b^ NIR, no intention to resign group, have only considered resigning once/never ^c^ Urban area: Tokyo 23 wards, Ordinance-designated cities, Core cities (cities with a population of 200,000 or more, excluding ordinance-designated cities), other cities, towns, or villages ^d^ Excluded junior residents

Ninety-two participants (26.0 %) were categorized into IR and 262 (74.0%) into NIR. The prevalence of IR was significantly higher in hospitals without mental health support than in hospitals with mental health support (47.2% vs. 19.7%, respectively, *P* < 0.001). Among IR medical residents, those who reported that communication frequency with their supervisors remained unchanged had slightly less intention to resign than those who reported an increase or decrease in communication frequency with their supervisors (21.8% vs.34.1% or 32.0%, respectively,* P* = 0.073). Furthermore, medical residents in the IR group who treated patients with COVID-19 compared to those who did not treat patients with COVID-19 had a higher intention to resign (28.3% vs. 18.3%, respectively, *P* = 0.07). Conversely, in the specialized clinical department, there was no statistically significant difference between the department of medicine and surgery in the IR group (33.0 % vs. 32.1 %, respectively, *P* = 0.901) (Table 1).

Table 2 shows the results of multivariate logistic regression analysis to investigate associated factors with the IR. As the years since graduation increased, the adjusted odds ratio showed an uptrend; the IR of those in PGY5 was 3.38 times higher than those in PGY1 (AOR = 3.38, 95% Cl [1.51-7.56], *P* = 0.003). Medical residents who treated patients with COVID-19 were also at higher risk of intent to resign than those who did not (AOR = 2.13, 95% Cl [1.08-4.18], *P* = 0.028). Furthermore, the absence of mental health support at their hospital was strongly associated with a high risk of IR (AOR = 2.95, 95% Cl [1.42-6.15], *P* = 0.004). Shortage of communication frequency with supervisors did not show any statistically significant association with the prevalence of IR.

**Table 2.** Multivariate Logistic Regression Analysis of Intention to Resign.

	AOR ^a^	95% Cl	*P-*value
Female	1.12	0.59-2.11	0.733
Postgraduate Year			
PGY 1	1[Reference]
PGY 2	0.97	0.44-2.17	0.946
PGY 3	1.26	0.55-2.85	0.585
PGY 4	1.94	0.83-4.51	0.124
PGY 5	3.38	1.51-7.56	0.003*
Sleeping hours	0.85	0.64-1.13	0.266
Number of hospital beds [n (%)]			
<300	1[Reference]	
300-700	0.79	0.34-1.83	0.579
≥700	1.33	0.54-3.32	0.537
Presence of mental health support at the hospital			
Yes	1[Reference]
No	2.95	1.42-6.15	0.004*
Do not know	1.51	0.83-2.76	0.175
Treated patient with COVID-19	2.13	1.08-4.18	0.028*
Communication frequency with supervising physicians			
Increased	1[Reference]
Decreased	0.81	0.34-1.93	0.634
No change	0.51	0.23-1.15	0.104
In the face of this COVID-19 pandemic, have you become interested or concerned about public health?			
Yes	1[Reference]
already been interested in	1.86	0.89-3.88	0.098
No	1.27	0.69-2.32	0.446

*P < 0.05 Abbreviations: AOR, adjusted odds ratio; Cl, confidence interval; PGY, postgraduate year; COVID-19, coronavirus disease 2019 ^a^ adjusted for gender, postgraduate year, sleeping hours, number of hospital beds, presence of mental health support at their hospital, treated patient with COVID-19, Communication frequency with supervising physicians, and interest in public health

## Discussion

Surprisingly, this study found that one in four medical residents wished to resign from their job during the COVID-19 pandemic. Medical residents who treated patients with COVID-19 and those at PGY5 with more experience had a higher risk of having the IR from their job compared to those at PGY1. Mental health support at their hospital was crucial in preventing medical residents from having the IR. Unlike previous reports ^(30), (31)^, communication frequency with supervisors does not seem to contribute enough to support medical residents during the COVID-19 pandemic.

According to a survey of Japanese physicians, the number of young physicians in their twenties who want to continue working at their current medical institution has decreased significantly after the COVID-19 pandemic (62.5% vs. 51.56%) ^(32), (33)^. Besides, previous studies have identified multiple factors influencing the intention to resign ^(34), (35), (36), (37)^. According to Kuriyama et al., highly resilient physicians were associated with a reduced risk of IR despite the perceived risk of infection or stigma ^(34)^. Resilience has been described as the process or ability to adapt positively despite difficult and threatening environments, and high resilience has been reported to prevent the occurrence of mental health disorders ^(34), (38)^. To strengthen the resilience of HCWs, providing professional mental health support at the organizational level is vital ^(34), (35), (39)^. Our results confirm these studies.

Younger HCWs are already known as a risk factor against the IR in multiple studies ^(8), (36)^. Conversely, PGY1 and PGY2 during their residency program are often well protected by the Medical Practice Act or the academic community ^(10), (40), (41)^. Takenoshita et al. mentioned that while PGY5 are becoming more confident in their clinical work, they are also expected to achieve academically, besides clinical chores. Thus, mentoring is crucial in supporting and managing medical residents at this stage ^(42)^. Mid-career young doctors, approximately around PGY 3-5, are often expected to be full-fledged physicians and forced to be independent, and they potentially fear the stigma of mental health disorders ^(33)^. Our study elucidated that PGY1 and mid-career young doctors must be intensively supported. Though the importance of communication was frequently reported, our study found that communication by supervisors alone may be insufficient. Considering the series of suicides committed by HCWs from multiple countries during the COVID-19 pandemic ^(43)^, this study concludes that the situation of HCWs was significantly difficult at this time, and special care for HCWs should be urgently established.

Multiple guidelines issued in the early stages of the COVID-19 pandemic emphasized the importance of self-care and organizational support by specialists like internists and psychologists for HCWs. These guidelines were issued by the British psychological society, the Inter-Agency Standing Committees (IASC), and the American psychological association (APA) ^(44), (45)^. The guidelines mentioned that establishing a mental health support desk was required at hospitals ^(46), (47)^. Even though attention had been paid to the mental health of HCWs, several studies reported that HCWs felt that mental health distress and burnout had worsened as time passed in the early stages of the COVID-19 pandemic ^(34), (48), (49)^. Our study also showed that one-third of medical residents are unconscious of their mental health conditions and do not recognize the availability of any mental support.

Our findings indicate that mental health support at the hospital could prevent medical residents from resigning from hospital work. Notably, mental support programs are needed for more experienced mid-career physicians. Under public health emergencies, such as the COVID-19 pandemic, wherein no HCWs have ever experienced, unexpected emotions could suddenly erupt. In this situation, important things to address include strengthening skills for appropriate self-control against reactions to anger, sadness, or confusion among HCWs ^(44)^. As observed in the great earthquake in Nepal in April 2015, depression and post-traumatic stress disorder reduced social cohesion ^(50)^. Even in medical institutions, poor mental state may negatively impact the medical team building of HCWs. It may be challenging to secure human resources promptly to establish mental health support during an emergency. However, it was emphasized that it is also essential to actively implement a strategy to value the healthcare professionals engaged in medical institutions daily; so safety ^(51)^ and sustainable medical services can be available for citizens during an emergency.

This study has six limitations. First, this study targeted Japanese medical residents with long-suffering temperaments. Therefore, it may not be adaptable to medical residents of other nationalities in other countries. Second, there is a possibility of misclassification bias because the Japanese tend to not be confident on speaking out about their IR. Third, recall bias could be considered because this study used self-report. Fourth, participants were recruited from a survey panel provided by a commercial research firm (PLAMED plus Co., Ltd, Tokyo, Japan). Therefore, selection bias is ineluctable since participants were not the representative. Fifth, our study showed the presence or absence of mental health support at the hospital. However, we have not confirmed that mental health support was actually active in each hospital. Sixth, this study was conducted during a second-time state of emergency. Therefore, this study did not include the number of residents who had the IR during the early COVID-19 pandemic periods. Consequently, the data may be collected and underestimated if a number of residents left the early COVID-19 pandemic periods.

In conclusion, one in four medical residents had the IR during the COVID-19 pandemic. Particularly, PGY5 and medical residents who treated patients with COVID-19 were found at risk of having IR. At times of crisis, such as the COVID-19 epidemic, establishing a mental health support system that targets high-risk residents is critical.

## Article Information

### Conflicts of Interest

None

### Sources of Funding

“Research for a medical care provision system based on preparations and responses to health crises such as new coronavirus infections” (Principal Investigator: Dr. Soichi Koike) from Japan’s Ministry of Health, Labour and Welfare. Grant Number 202006026A. The grant agency was not involved in data collection; analysis or interpretation; trial design; resident recruitment; or any aspect pertinent to the study.

### Acknowledgement

This study was supported by the Ministry of Health, Labour and Welfare of Japan, with grant number 202006026A, “Research for a medical care provision system based on preparations and responses to health crises such as new coronavirus infections” (Principal Investigator: Dr. Soichi Koike). We gratefully acknowledge Soichi Koike, MD and PhD (Division of Health Policy and Management, Centre for Community Medicine, Jichi Medical University) for professional advice. We thank Kenji Hira, MD, PhD (PLAMED plus Co., Ltd, Tokyo, Japan) for professional advice in conducting this study. We also appreciate Aliza KC Bhandpsychologial distress, RN, MPH; Kimi Estela Kobayashi-Cuya, MHSc, PhD; Haruna Nishio and Mrs. Akiko Yamazaki, (Graduate School of Public Health and Clinical Epidemiology St. Luke’s International University) for their helpful advice.

### Author Contributions

All authors conceived the ideas; T.M and S.O collected, statistically analyzed, and interpreted the data; T.M and S.O led the writing of the manuscript; All authors participated in critically reviewing the study. Critical revision of the manuscript for important intellectual content; S.O obtained funding; S.O supervised the study. All authors read and approved the final manuscript.

### Approval by Institutional Review Board (IRB)

This study was conducted by the Declaration of Helsinki and approved by the Research Ethics Committee of St. Luke’s International Hospital (approval code: 20-R216). Informed consent was through a form on the questionnaire website.

## Supplement

Supplementary Material Impact of COVID-19 pandemic for medical residents -questionnaire.
