# Supplementary material for: Factors Associated with Intention to Resign among Medical Residents during the COVID-19 Pandemic in Japan: A Cross-sectional Study
Source: JMA J. 2023 Jul 4;6(3):292–9. doi: 10.31662/jmaj.2023-0004 (PMC10407254; doi:10.31662/jmaj.2023-0004)
Supplement: Supplementary Material — Impact of COVID-19 pandemic for medical residents -questionnaire. [file 2433-3298-6-3-0292-s001.pdf]

Questionnaire for Impact of COVID-19 pandemic on medical residents

*Partly undisclosed

Q1. Please choose your age:

1. 24-25 2. 26-30 3. 31-35 4. 36-40 5. 41-45 6. 46 or over

Q2. Please choose your gender:

1. Male 2. Female

Q3. Please choose the year of medical training you are in:

(PGY: Post graduate year)

1. PGY1 2. PGY2 3. PGY3 4. PGY4 5. PGY5

Q4. Where is your hospital located?

1. Urban area 2. Other

Q5. Please choose the number of hospital beds at your workplace:

1. <100 2. 100-299 3. 300-499 4. 500-699 5. 700- 899 6. ≧900

Q6. Please write down your average working hours in the last week

( ) hours / week

Q7. Please write down your recent average sleeping hours per day.

( ) hours / day

Q8. What is your residency specialty? Excluded junior residents

1. Internal Medicine 2. Surgery 3. Pediatrics
4. Obstetrics and Gynecology 5. Other

Q9. Have you been engaged in medical care and logistical support for patients with COVID-19 (including suspected cases of fever in the outpatient clinic)?

*Logistical support includes working at a hotel for the treatment of patients with mild symptoms, supporting the public health center, preparing guidelines in your hospital, and so on.

1. Yes 2. No

Q10. If you answered yes to the previous question, please tell us the number of patients with

COVID-19 (including suspected cases of fever in the outpatient clinic) you have treated in the last month.

Approximately (   ) patients/month

Q11. How has COVID-19 affected your communication with your supervisor?

1. Increased   2. Decreased   3. No change

Q12. Do you have a consultation service that provides mental health support at your hospital?

1. Yes   2. No   3. Do not know

Q13. Have you ever wanted to quit the hospital due to the stress of dealing with patients with COVID-19?

1. I never thought about it   2. I have thought about it only once.

3. I sometimes have thought about it   4. I am thinking about it right now

Q14. In the face of this COVID-19 pandemic, have you become interested or concerned about public health?

1. Yes   2. Already been interested   3. No
